# Bioassays for the evaluation of the attractiveness of attractive targeted sugar bait (ATSB) against *Anopheles* mosquitoes in controlled semi-field systems

**DOI:** 10.1186/s13071-024-06653-3

**Published:** 2025-02-04

**Authors:** Frank S. C. Tenywa, Ummi A. Kibondo, Julian Entwistle, Osward Dogan, Mapipi Haruna, Restuta P. Phisoo, Jason Moore, Jane J. Machange, Haji Makame, Frederic Tripet, Pie Müller, Mathias Mondy, Derric Nimmo, Jennifer C. Stevenson, Sarah J. Moore

**Affiliations:** 1 Vector Control Product Testing Unit, Environmental Health and Ecological Sciences, Ifakara Health Institute, P.O. Box 74, Bagamoyo, Tanzania; 2 Swiss Tropical and Public Health Institute, Kreuzstrasse 2, 4123 Allschwil, Switzerland; 3 University of Basel, Petersplatz 1, 4001 Basel, Switzerland; 4 Liverpool School of Tropical Medicine, The Innovative Vector Control Consortium, Pembroke Place, Liverpool, L3 5QA UK; 5 Centre for Applied Entomology and Parasitology, School of Life Sciences, Keele University, Newcastle, Staffordshire ST5 5BG UK; 6 Nelson Mandela African Institute of Science and Technology (NM-AIST), P.O. Box 447, Tengeru, Tanzania

**Keywords:** Attractive sugar bait, Attractive targeted sugar bait, *Anopheles gambiae *s.s., *Anopheles funestus *s.s., Olfactory attraction, Sugar feeding

## Abstract

**Background:**

Sugar feeding is an essential aspect of mosquito biology that may be exploited for mosquito control by adding insecticides to sugar attractants, so-called ‘attractive targeted sugar baits’ (ATSBs). To optimize their effectiveness, ATSB products need to be maximally attractive at both short and long range and induce high levels of feeding. This study aimed to assess the attractiveness and feeding success of *Anopheles* mosquitoes exposed to attractive sugar baits (ASBs).

**Method:**

Experiments were conducted in 2 × 5 × 2-m cages constructed within the semi-field systems (SFS) at Ifakara Health Institute, Bagamoyo, Tanzania. Male and female *Anopheles gambiae* s.s. and *An. funestus* s.s. mosquitoes were exposed to either 20% sucrose or different ASB station prototypes produced by Westham Co. in either (1) no-choice experiments or (2) choice experiments. Mosquitoes were exposed overnight and assessed for intrinsic or relative olfactory attraction using fluorescent powder markers dusted over the ASB stations and 20% sucrose and for feeding using uranine incorporated within the bait station and food dye in 20% sucrose controls.

**Results:**

Both male and female *An. gambiae* and *An. funestus* mosquitoes were attracted to the ASBs, with no significant difference between the sexes for each of the experiments conducted. Older mosquitoes (3–5 days) were more attracted to the ASBs (OR = 8.3, [95% CI 6.6–10.5] *P* < 0.001) than younger mosquitoes (0–1 day). Similarly, older mosquitoes responded more to 20% sucrose (OR = 4.6, [3.7–5.8], *P* < 0.001) than newly emerged *Anopheles*. Of the four prototypes tested, the latest iteration, ASB prototype v1.2.1, showed the highest intrinsic attraction of both *Anopheles* species, attracting 91.2% [95% CI 87.9–94.5%]. Relative to ATSB v1.1.1, the latest prototype, v.1.2.1, had higher attraction (OR = 1.19 [95% CI 1.07–1.33], *P* < 0.001) and higher feeding success (OR = 1.71 [95% CI 1.33–2.18], *P* < 0.001).

**Conclusions:**

Data from these experiments support using ASBs v1.2.1, deployed in large-scale epidemiological trials, as it is the most attractive and shows the highest feeding success of the Westham prototypes tested. The findings indicate that future bioassays to evaluate ATSBs should use mosquitoes of both sexes, aged 3–5 days, include multiple species in the same cage or chamber, and utilize both non-choice and choice tests with a standard comparator.

**Supplementary Information:**

The online version contains supplementary material available at 10.1186/s13071-024-06653-3.

## Background

Since the early 2000s, malaria cases and deaths were significantly reduced in sub-Saharan Africa because of the widespread deployment of insecticide-treated nets (ITNs) and indoor residual spraying (IRS) as well as early diagnosis and treatment with effective antimalaria drugs [1]. However, sustained selection pressure on mosquitoes from insecticides used in agriculture and those used on ITNs and IRS, especially pyrethroids, has led to widespread insecticide resistance among mosquito malaria vectors [2], undermining malaria control [3, 4]. To maximise and sustain the impact of insecticide-based vector control, it is necessary to utilise insecticide-resistance management (IRM) techniques that preserve or prolong the susceptibility of mosquitoes to insecticides. The World Health Organisation (WHO) recommends regular and frequent monitoring of vector susceptibility to insecticides to inform judicious selection of insecticides for vector control, combined with IRM strategies that ensure that single classes of insecticides are not used everywhere and over consecutive years [5, 6]. Deploying tools involving novel insecticides with alternative modes of action alongside or instead of pyrethroid-based interventions can mitigate the potential impact of pyrethroid resistance. Such tools are more likely to be impactful against insecticide-resistant mosquitoes if the active ingredients they contain have not been previously used in vector control [5] or in agriculture where *Anopheles* larvae are exposed to pesticide run-off [7–12].

As well as the evolution of resistance in mosquitoes to insecticides used for ITNs and IRS, the potential of these tools to control malaria is limited by typically being deployed indoors. Indoor deployment also limits the range of insecticides that can be used; active ingredients used must meet stringent safety requirements because of the risk of people contacting the insecticide, especially when used for treating ITNs. Furthermore, the range of chemicals that can be used may be constrained by the specific properties required. For example, chemicals used to treat ITNs must be of low water solubility to withstand washing [13, 14] and insecticides for IRS need to remain efficacious when applied to walls of different compositions [15].

The application of insecticides via oral ingestion has been used for fly control for over 100 years [16] and more recently for the control of mosquitoes [17] and sandflies [18]. Insecticidal sugar baits that attract mosquitoes are called attractive targeted sugar baits (ATSBs) [19] and exploit the reliance of most Diptera on carbohydrates for energy to maximize their survival and reproductive success [20]. Both male and female mosquitoes feed on sugar [21]. Therefore, an intervention that targets males [22] via their sugar feeding behaviour will potentially have a greater impact than those that only target female mosquitoes while host-seeking (ITNs) or resting after a blood meal (IRS).

Applying insecticides for uptake via the oral route has the potential to significantly increase the diversity of compounds that may be used in public health for several reasons: application is targeted and used in a format minimizing human and animal contact, meaning that compounds may not need to meet the stringent safety needs of those on nets; the insecticides are presented in solution so water-soluble compounds (unsuitable for ITNs) may be used; and oral ingestion means that insecticides with novel modes of action that do not function by cuticular contact could be used. Many active ingredients used in ATSBs have been shown to be effective in killing mosquitoes and include a variety of insecticide classes, as described in Table 1.

**Table 1:** List of active ingredients used in ATSBs

Insecticide class	Insecticide	Target mosquitoes	(Study examples)
Carbamates	Bendiocarb	*Culex pipiens molestus*	[23]
Organophosphates	Malathion and pirimiphos-methyl	*Aedes aegypti* and *Culex pipiens molestus*	[23, 24]
Phenylpyrazoles	Fipronil	*Culex quinquefasciatus, Anopheles quadrimaculatus* and *Aedes taeniorhynchus*	[25]
Pyrethroids	Permethrin, bifenthrin, α-cypermethrin, λ-cyhalothrin and d-phenothrin	*Culex quinquefasciatus, Anopheles quadrimaculatus* and *Aedes taeniorhynchus* *Culex pipiens molestus*	[23]
Neonicotinoids	Dinotefuran, imidacloprid and thiamethoxam	*Culex quinquefasciatus, Aedes aegypti Anopheles quadrimaculatus* and *Aedes taeniorhynchus*	[25, 26]
Pyrroles	Chlorfenapyr	*Anopheles gambiae, Anopheles arabiensis* and *Culex quinquefasciatus*	[27]
Spinosyns	Spinosad	*Culex quinquefasciatus, Anopheles quadrimaculatus* and *Aedes taeniorhynchus*	[24]
Endectocides	Ivermectin	*Culex quinquefasciatus, Anopheles quadrimaculatus* and *Aedes taeniorhynchus*	[24]
Juvenile hormone analogues	Pyriproxyfen	Adult and larvae *Aedes albopictus*	[28]
Microbial insecticides	*Bacillus* spp., *Pseudomonas* spp. and *P. stewartii Metarhizium anisopliae* spp.	*Anopheles gambiae s.s., Anopheles arabiensis* and *Aedes aegypti*	[29, 30]
Botanicals	Garlic oil, encapsulated in ß-cyclodextrin and eugenol	*Aedes albopictus, Aedes aegypti*, *Culex quinquefasciatus*, *Anopheles quadrimaculatus* *Aedes atlanticus*, *Aedes infirmatus*, *Culex nigripalpus Anopheles crucians*, *Uranotaenia sapphirina*, *Culiseta melanura* and *Culex erraticus*	[31, 32]
RNA based technologies	RNA interference (RNAi) and siRNA	*Aedes aegypti, Aedes albopictus*, *Anopheles gambiae* and *Culex quinquefasciatus*	[33, 34]

Mosquitoes can distinguish between different sugar sources using olfactory cues [35–37]. Therefore, in addition to the killing agent, ATSBs are augmented with olfactory cues to attract mosquitoes and also incorporate sugar to stimulate feeding. For decades, since the first test of sugar baits as targets for mosquito control [24], multiple studies have explored the most attractive lures from plant sources [36–42], and some attractive floral blends have been developed [36, 43]. The developed lures have been used to make ATSBs by adding a toxin to the sugar solution and deploying the toxic bait through simple means such as soaking cotton wool [27, 42, 44] or spraying plants [28, 31, 45–47]. Many of the ATSBs evaluated have a short shelf life, may impact non-target organisms or both. However, Westham Co. has developed a long-lasting, weather-resistant ATSB with a long shelf life designed for deployment in programmatic vector control [48]. These bait stations contain the active ingredient dinotefuran (a neonicotinoid class insecticide), antibacterial and antifungal additives and date syrup. The bait is contained within a protective membrane that covers and protects the baits from UV damage, rain and dust but allows mosquitoes to feed through the pores. The membrane serves as a barrier to pollinators and other non-target insects that do not have piercing mouthparts [49].

The Westham ATSB stations were shown to successfully reduce entomological inoculation rates (EIRs) in field trials in Mali [48], and, based on this success, their efficacy is being investigated both entomologically and epidemiologically in large-scale randomised controlled trials in other parts of sub-Saharan Africa [50]. Whilst the study in Mali demonstrated a marked impact against *An. gambiae *s.l. in the area, no published studies to date have investigated the response of other mosquitoes endemic to sub-Saharan Africa, such as *Anopheles funestus*, which play a key role in malaria transmission in many parts of central, east and southern Africa. The efficacy of the ATSBs against mosquitoes of different ages and comparisons of attraction and feeding on ATSBs between males and females have yet to be studied. New prototypes of the Westham ATSBs have been developed since the Mali field trial. To identify the most efficacious version with potential public health value, the intrinsic and relative attraction and feeding success of these different ATSB iterations need to be evaluated in a controlled setting.

This article presents results from controlled semi-field studies in Tanzania, assessing whether mosquito age, sex and species affect mosquito attraction and feeding success on Westham bait stations (without toxin, i.e. ASBs). The study also evaluated how these factors influenced responses to three ASB prototypes in both choice and non-choice experiments, determining the intrinsic and relative preferences of *Anopheles* mosquitoes for different versions of the ASB stations.

The findings from this study were used to develop a standardised bioassay for use in future semi-field evaluations of mosquito attractiveness and feeding success of ASBs and ATSBs.

## Methods

The main aims of this study were to: (i) compare the attraction and feeding success of malaria vectors on different prototypes of ASB stations and (ii) develop a standardised SOP for future studies to evaluate ATSBs in a semi-field system (SFS).

The term “attraction” in this study refers to mosquito landing on the ASB regardless of whether it is feeding on the ASB or not.

In this study, ASB stations v1.0, v1.1.1, v1.1.2 and v1.2.1 were used. ASB station v1.0 was an earlier prototype without the Bitrex taste deterrent, ASB stations v1.1.1 and v1.1.2 were more advanced prototypes incorporated into the Bitrex taste deterrent, while the ASB station v1.2.1 was the most advanced prototype with the highiest throughput and also contained Bitrex. The later version was used in large-scale epidemiological trials in Kenya, Zambia and Mali.

The specific study objectives were to:

(i) Determine whether the mosquito age affects intrinsic mosquito attraction of bait stations and a standardised comparator, 20% sucrose, prepared by dissolving 20 g sucrose into 100 g solution;
(ii) Determine the intrinsic mosquito olfactory attraction of different prototypes of ASB stations and of a standardised comparator, 20% sucrose;
(iii) Compare the relative attractions of different ASB station prototypes against each other;
(iv) Determine the intrinsic feeding success of mosquitoes on different ASB station prototypes;
(v) Investigate any differences between attraction and feeding success between male and female mosquitoes and between two mosquito species, *An. gambiae* and *An. funestus;*
(vi) Develop a standardised SOP for future studies to evaluate ATSBs in a SFS.

### Study site and design

Experiments were conducted in the SFS at Ifakara Health Institute, Bagamoyo, Tanzania, 6°8′S, 30°37′E. Annual rainfall in Bagamoyo district ranges between 800 and 1000 mm, with temperatures between 22 and 33 °C and mean relative humidity of 73%. The SFS measured 29 m × 21 m and was built from a fabricated greenhouse frame set on concrete and modified to make two compartments with a central corridor and an opaque polyethylene roof for rain protection. Netted sides allowed for acclimatisation with ambient conditions [51]. To evaluate the intrinsic mosquito olfactory attraction and feeding success (objectives i, ii and iv), ‘no-choice’ experiments were conducted in the SFS, where the stations or sucrose solutions were deployed alone in 2 × 5 × 2-m cages erected in separate chambers. For investigations of the relative attraction of different prototypes of ATSB stations (objective iii), ‘choice’ experiments were used, where two stations were deployed in the same cage (Table 2). In both experimental setups (no-choice and choice experiments), mosquitoes were given water delivered through a ball of cotton wool in a bowl placed 1 m away from the bait station to enable mosquitoes access moisture to prevent them from desiccation.

**Table 2:** Experimental setup for no-choice and choice experiments in 2 × 5 × 2-m cages in the semi-field system

Experimental design				
	*No choice*	*No choice*	*Choice*	*No choice*
Objective	1. Intrinsic attraction of 0–1- and 3–5-day-old mosquitoes	2. Intrinsic attraction to ASBs	3. Relative attraction to ASBs	4. Feeding success on ASBs
Experimental arms	ASB station v1.1.1 with 0–1-day-old mosquitoes ASB station v1.1.1 with 3–5-day-old mosquitoes 20% sucrose with 0–1-day-old mosquitoes 20% sucrose with 3–5-day-old mosquitoes	ASB station v1.2.1 ASB station v1.1.1 ASB station v1.1.2 20% sucrose	ASB station v1.1.1 vs ASB station v1.1.2 ASB station v1.1.1 vs ASB station v1.2.1 ASB station v1.1.2 vs ASB station v1.2.1	ASB station v1.2.1 ASB station v1.1.1 ASB station v1.1.2 20% sucrose
Outcomes	Intrinsic and relative attraction			Intrinsic feeding success
Mosquito species	*Anopheles gambiae *s.s. (Kisumu)	*Anopheles gambiae s.s. *(Kisumu) *Anopheles funestus *s.s. (FUMOZ)		
Mosquito sex	Male and female			
Mosquito age	0–1 day old, blood and sugar naïve 3–5-days blood naïve and sugar-starved for 6–8 h	3–5-days blood naïve and sugar-starved for 6–8 h		
Mosquitoes per replicate	50 per sex per species			
Replicates per arm	8–14			
Exposure time	12 h			

All experiments in the two experimental setups (no-choice and choice experiments) released both sexes of the species at the same time within a cage. Experiment 1 used males and females of *An. gambiae*, while in experiments 2–4 males and females of both *An. gambiae* and *An. funestus* were released simultaneously. To address objective v, responses of different sexes and species to the bait stations were statistically analysed.

### Mosquitoes

All experiments used insectary-reared insecticide-susceptible *An. gambiae* s.s. (Kisumu) and *An. funestus* s.s. (FUMOZ). The mosquitoes were reared following MR4 guidelines [52] at temperatures of 27 °C ± 2 °C, 75% ± 10% relative humidity and ambient 12:12 light:dark cycle at the Vector Control and Product Testing Unit, Ifakara Health Institute (IHI), Bagamoyo. Larvae were fed with Tetramin® fish food (Tetra Werke Co., Melle Germany), while adults were maintained with 10% m/v sucrose solution. For egg laying, female adult mosquitoes were fed cow blood using a membrane feeding technique.

### Preparation of ASBs and controls

The standard comparator used in this study was determined prior to carrying out the experiments evaluating ASBs. A series of no-choice experiments comparing different concentrations of sucrose and fructose demonstrated that 20% sugar solution was the most attractive with no difference between sucrose or fructose (see supplementary materials for full experimental details and results, SOM Table 1). Sucrose at 20% *m*/*v* was used for all experiments because of its availability and lower price.

**Fig. 1:**
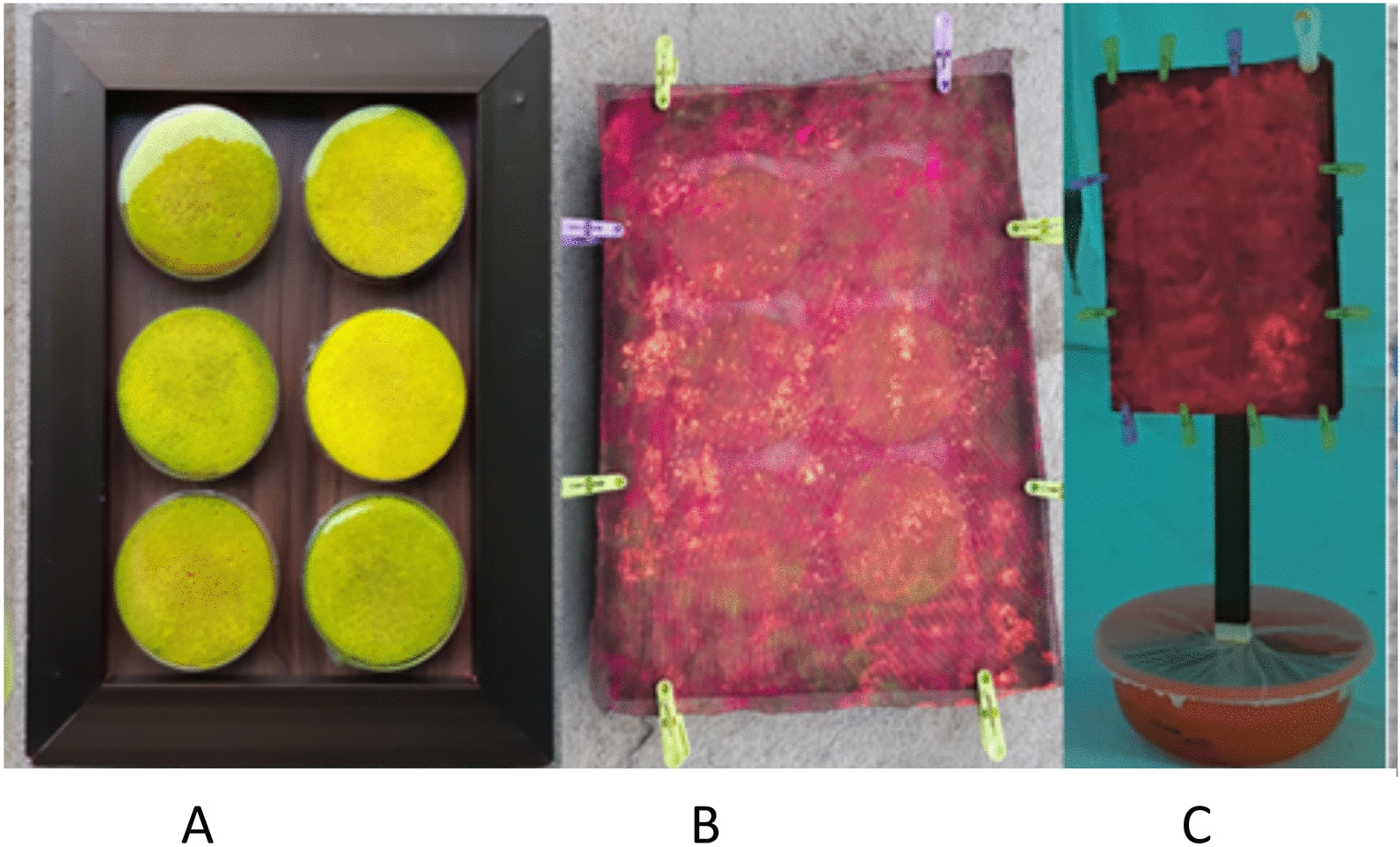
Battery of sugar solution-filled Petri dishes. **A** Standardised control 20% sucrose-filled Petri dishes labelled with uranine and covered with clingfilm on an aluminium tray. **B** Petri dishes overlaid with electrostatic black gauze/netting (EN) dusted with fluorescent powder. **C** Sugar delivery system (either a tray of Petri dishes or bait station covered with EN) and erected within a net-covered dish

The sugar comparator was prepared by dipping a cellulose sponge foam disc (O-Cel-O, Scotch Brite, St. Paul, MN, USA) into 20% sucrose solution containing 0.5% *v*/*v* food dye (Carmoisin) (for visualisation of fed mosquitoes) and placing the disc into Petri dishes (Sigma Aldridge®, Burlington, MA, USA) of approximately 11 cm diameter and 90 ml volume. The Petri dishes were overlaid with one layer of cling film pierced in a 0.5-cm grid with sterile pins to produce accessible, small droplets of sugar solution without excessive leaking of the solution on the surface of the cling film (Fig. 1A). For experiments determining intrinsic and relative attraction of mosquitoes to ASBs or sucrose, black electrostatic netting (EN) (PollenTec, Phoenix, AZ, USA) was installed above the cling film on the Petri dishes containing sucrose or the bait station membranes. The netting was positioned 2 mm above both the controls and ASBs to prevent contact of the EN with the surface below. Fluorescent dust was applied to the overlaid EN to enable marking of those mosquitoes that landed on the bait looking for a sugar meal [53] (Fig. 1B). For choice experiments, different-coloured fluorescent dusts were used to distinguish the responses to the specific station used. The trays of Petri dishes or ASBs were attached to a pole and set in a net-covered dish, which prevented mosquitoes from feeding on any solution that leaked into the dish (Fig. 1C). As with the controls, the ASBs were labelled with a fluorescent marker (0.8% *w*/*v* uranine fluorescent dye, Sigma Aldridge®) for visualisation of fed mosquitoes. For studies of feeding success, no EN was overlaid to allow mosquitoes to readily access sugar from the Petri dish or bait station without an extra barrier.

### Experimental procedures

For each experiment, blood-naïve and either sugar-starved for 6–8 h or sugar-naïve mosquitoes (for those newly emerged) were released at 18:00 h within the cages and collected at 06:00 h the following morning using a Prokopack aspirator [54]. Following collection, mosquitoes were taken to the insectary, killed in a freezer and inspected. A light microscope using a UV light torch was used to detect fluorescent powder on the body surface as an indicator of attraction for experiments 1–3, and a fluorescent microscope (Germany made Leica DMLS fluorescence microscope) was used to visualise fluorescent sugar meals in the abdomen as an indicator of feeding success [55]. Mosquitoes were scored by species, sex and marking status for each experiment and study arm. The specific setup and methodology of each experiment are detailed below.

#### Determination of whether the mosquito age affects intrinsic mosquito attraction of bait stations or a standardised comparator, 20% sucrose

Before investigating the effect of mosquito age on the attraction of ASBs, an experiment was run to compare the attractiveness of two different early ASB prototypes/stations v1.0 and v1.1.1, available at the time, to newly emerged *An. gambiae *s.s. to decide which station to use for further investigation (see supplementary materials, Table 2 SOM 1). Intrinsic and relative attraction experiments demonstrated greater attraction of young mosquitoes to the more recent version, ASB station v1.1.1, than to ASB station v1.0, so ASB station v1.1.1 was selected to investigate mosquito age, and ASB station v1.0. was not used in any subsequent experiments.

To determine the effect of mosquito age on their attraction to ASBs and therefore determine which age cohorts to use in subsequent experiments, assays were only conducted using the insecticide-susceptible *An. gambiae *s.s. Kisumu, a standard laboratory strain used in several facilities worldwide. ASB station v1.1.1 and the 20% sucrose standardised comparator were placed in four separate cages (2 m × 5 m × 2 m each) within the SFS chambers. Two cages contained the ASB station, and two contained the 20% sucrose comparator. In each cage, 50 male and 50 female *An. gambiae *s.s. mosquitoes were released. The mosquitoes were either newly emerged (0–1 days) sugar and blood-naïve or 3–5 days old, blood-naïve and sugar-starved for 6–8 h. One age cohort was used per cage and treatment, resulting in four experimental arms. The experiment was replicated over 8 nights in November 2020. Each night, the two age cohorts were rotated between cages to control for potential locational biases (e.g. proximity to lights or competing sources of kairomones). The results of this study were used to determine the optimal mosquito age for use in subsequent experiments.

#### Determination of the intrinsic mosquito attraction of different prototypes of ASB stations and of a standardised control, 20% sucrose

**Fig. 2:**
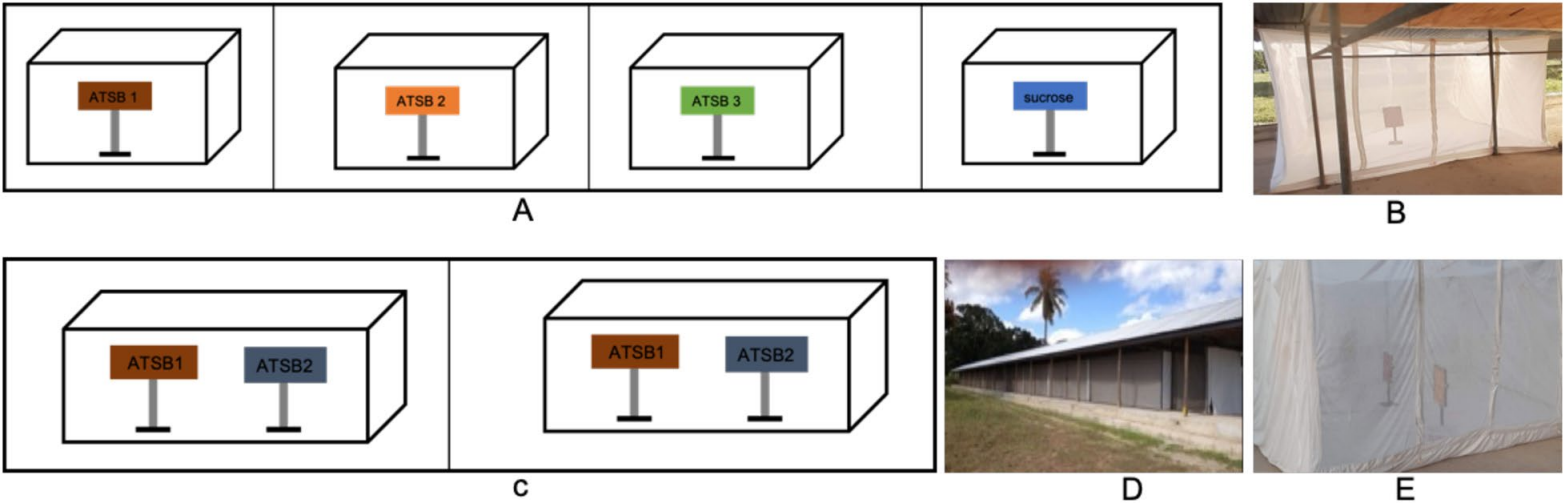
Schematic diagrams and images for intrinsic and relative attraction experimental setup. **A** ASB stations or 20% sucrose solution placed into separate cages for measuring the intrinsic attraction of the bait stations. **B** Image of the cage with a bait station for measuring an intrinsic attraction. **C** Two ASB stations placed in the same cage to measure the relative attraction of the bait stations. **D** Image of the Ifakara ambient chamber test in which the 2 × 5 × 2-m cages were placed for measuring the intrinsic and relative attractions. **E** Image of a cage with two bait stations for measuring relative attraction

The intrinsic olfactory attraction of the ASB stations was assessed under semi-field conditions using no-choice experiments in the 2 × 5 × 2-m cages within the SFS. Each cage was randomly assigned one treatment: ASB station v1.1.1, ASB station v1.1.2, ASB station v1.2.1 or 20% sucrose standard comparator (Fig. 2A and B). The assignment of a specific treatment/control to a cage was changed after every replicate (experimental night) to mitigate locational bias. In each cage, 50 females and 50 males of each species (*An. gambiae *s.s. and *An. funestus *s.s. to make a total of 200 mosquitoes) aged 3–5 days old, blood-naïve and sugar-starved for 6–8 h were released into each cage at the same time. Per arm, 12 replicates (experimental nights) were carried out between January and June 2021.

#### Comparison of the relative attraction of different prototypes of ASB stations against each other

Three different bait station prototypes were compared in two dual-choice test experiments, conducted within 2 × 5 × 2-m cages in the SFS. Before selecting the prototypes for comparison, an initial study was performed in these cages to compare the attractiveness of ASB station v1.0, ASB station v1.1.1 (with bait) and a blank ASB station (v1.1.1 without bait) (see SOM 1). The results showed that ASB station v1.1.1 with bait was significantly more attractive than both the blank ASB station and ASB station v1.0. Based on these findings and those from the intrinsic attraction no-choice experiment (Experiment 2), ASB station v1.0 was excluded from further experiments (Experiments 3–4).

To assess relative attraction of three prototypes, the following comparisons were conducted, with each pair assigned to a different cage:

(i) ASB station v1.1.1 vs ASB station v1.1.2
(ii) ASB station v1.1.1 vs ASB station v1.2.1
(iii) ASB station v1.1.2 vs ASB station v1.2.1.

The higher version number represents more advanced iterations of the ASB stations. In each test, the stations were placed 1 m apart within the chamber (Fig. 2C and E). Fifty 3–5-day-old, blood-naïve and sugar-starved female and male *An. gambiae* s.s. and *An. funestus* s.s. were released into the chambers. Twelve replicates (nights) per comparison were conducted between July and November 2021.

#### Determination of the intrinsic feeding success of mosquitoes on different prototypes of ASB stations

ASB station v1.1.1, ASB station v1.1.2, ASB station v1.2.1 and 20% sucrose standard comparator were each placed into the 2 × 5 × 2-m cages. For these experiments, the electrostatic netting was not installed in front of the bait stations or the comparators, so that mosquitoes had free access to feed on the bait. Each night, 50 female and 50 male *An. gambiae* s.s. and *An. funestus* s.s. mosquitoes, which were 3–5 days old, blood-naïve and sugar-starved, were released into each cage each night. Eight replicates per arm were performed, and the experiment was conducted between April and May 2022.

#### Investigation of differences in attraction and feeding success between male and female mosquitoes and between the two mosquito species, *An. gambiae* and *An. funestus*

Data analyses for all experiments investigated the effect of mosquito sex on attraction and feeding. For those experiments that included the two different species together, additional analyses were carried out to investigate whether the mosquito species *An. gambiae* s.s. and *An. funestus* s.s. responded differently (attraction and feeding).

#### Development of a standardised SOP for future studies to evaluate ATSBs in a semi-field system

The SOP for a standardised bioassay for further evaluations of the attractiveness of ATSBs to *Anopheles* mosquitoes and bait-feeding success in a controlled semi-field environment was developed as described in SOM 2.

### Data analysis

For each experiment, descriptive analysis was performed to compare the arithmetic mean percentage across the different bait stations and the standard comparator in terms of the number of mosquitoes attracted (landed on and marked) and fed. Binomial logistic regression was performed separately for each experiment to compare the following:

Experiment 1 (no-choice): the proportion of released mosquitoes aged 0–1 days vs those aged 3–5 days attracted to ASB v1.1.1 and the standard comparator.

Experiment 2 (no-choice): the proportion of released mosquitoes that landed on each of the different iterations of the ASBs and the standard comparator.

Experiment 3 (choice): the proportion of released mosquitoes attracted to (i) ASB v1.1.1 vs ASB v1.1.2; (ii) ASB v1.1.1 vs ASB v1.2.1; (iii) ASB v1.1.2 vs ASB v1.2.1;

Experiment 4 (no-choice): the proportion of released mosquitoes that fed on each of the different iterations of the ASBs and the 20% sucrose comparator.

For each of these experiments, the responses of females were compared to males. In experiments 2–4, where both *An. gambiae* and *An. funestus* were used, the responses of these two species were also compared.

All statistical models included bait station type (ASB or sucrose where applicable), mosquito age, species, sex and experimental day as fixed categorical variables. Additionally, an interaction term for the bait station type and mosquito species was introduced in the model to determine whether the two species responded differently to each version of the bait station.

For all analyses, odds ratios and 95% confidence intervals (95% CI) were estimated from the regression models. Pairwise comparisons of the log odds were performed to analyse the differences between bait stations. All analyses were performed using STATA 16 software (StataCorp LLC, College Station, TX, USA).

## Results

### Determination of whether mosquito age may affect intrinsic mosquito attraction of bait stations or a standardised comparator, 20% sucrose

**Fig. 3:**
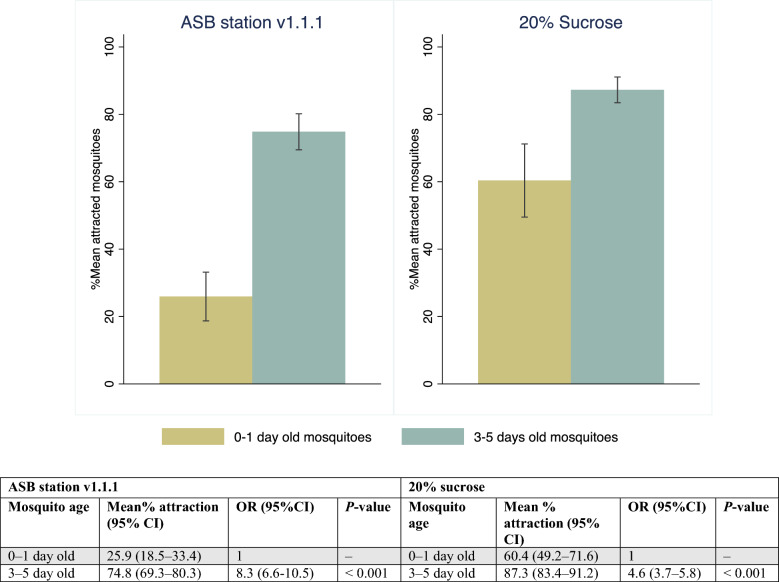
Olfactory attraction of 0–1-day-old compared to 3–5-day-old *Anopheles gambiae *s.s. when exposed to ASB station v1.1.1 (Westham bait station) and 20% sucrose in 2 × 5 × 2-m cages. The table below provides the odds ratios (ORs) and *P*-values comparing the response of each age to ASB station and sucrose. OR: odds ratio, 95% CI: 95% confidence interval

The attraction of older *An. gambiae* s.s. mosquitoes (3–5 days old) to ASB station v1.1.1 was significantly higher than that of younger (0–1 days) mosquitoes (Fig. 3). Approximately 50% more 3–5-day-old *An. gambiae* s.s. were attracted to ASB station v1.1.1 [74.8% (95% CI 69.3–80.3)] than the 0–day old mosquitoes [25.9% (95% CI 18.5–33.4)] (Fig. 3) regardless of sex (see Table 3 SOM1 for response by sex). A similar trend was observed for the 20% sucrose standard comparator, where > 80% of 3–5-day-old *An. gambiae* s.s. were attracted to 20% sucrose [87.3% (95% CI 83.4–91.2)] compared to 60.4% (95% CI 49.2–71.6) of the 0–1-day-old *An. gambiae* s.s. (Fig. 3).

### Determination of the intrinsic mosquito attraction of different prototypes of ASB stations and of a standardised control, 20% sucrose

**Fig. 4:**
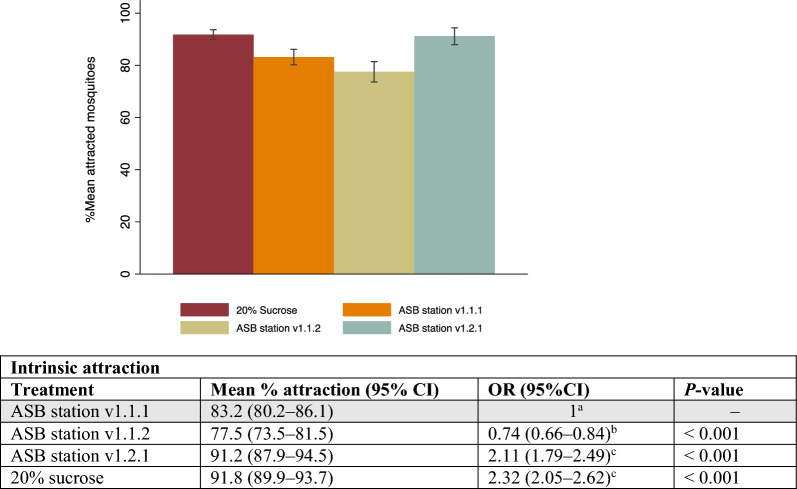
Intrinsic attraction of different ASB stations and sucrose for 3–5-day-old mosquitoes in 2 × 5 × 2-m cages. The table below provides the odds ratios (ORs) and *P*-values comparing the response of mosquitoes to each station/sugar. OR: odds ratio, 95% CI: 95% confidence interval. Differing superscript letters are significantly different from each other. The same superscript letters are not significantly different from each other

The attraction of mosquitoes to all baits tested in no-choice experiments was close to or exceeded 80% (Fig. 4). The different iterations of the Westham bait stations showed an increasing ability to attract mosquitoes, with the latest prototype, ASB station v1.2.1, attracting the highest proportion of mosquitoes, 91.2% (95% CI 87.9–94.5%), compared to ASB station v1.1.1, 83.2% (95% CI 80.2–86.1%) (Fig. 4). This was comparable to the attraction observed in the 20% sucrose comparator.

When all ASB stations were analysed together, there was no significant difference in response between *An. gambiae *s.s. and *An. funestus *s.s. However, in the individual bait analyses, ASB station v1.2.1 was marginally less attractive to *An. funestus *s.s. (89.9%, 95% CI 83.9–96.0%) than to *An. gambiae *s.s. (92.4%; 95% CI 89.3–95.5%); OR = 0.70, 95% CI 0.52–0.94, *P* = 0.018) (Table 4 SOM 1).

Similarly, when all bait stations were considered together, there was no difference in the response between mosquito sexes. However, ASB station v1.1.2 was found to be more attractive to female mosquitoes (80.8%, 95% CI 75.7–86.0%) than males (74.3%, 95% CI 68.1–80.5%; OR = 0.67, 95% CI 0.55–0.82, *P* < 0.001) when analysed by station version (Table 4 SOM 1).

### Comparison of the relative attraction of different prototypes of ASB stations against each other

**Fig. 5:**
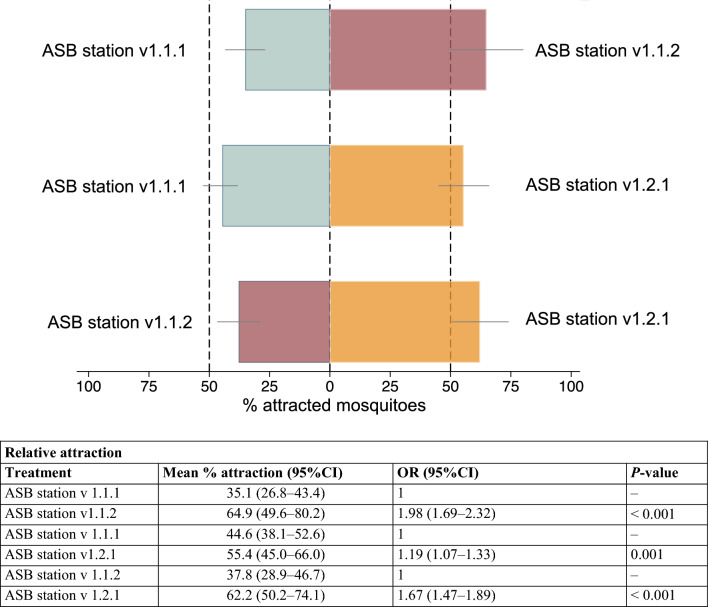
Relative attraction of ASB stations in choice tests for 3–5-day-old mosquitoes in 2 × 5 × 2-m cages. The table below provides the odds ratios (ORs) and *P*-values comparing the response of mosquitoes to the different ASB stations. OR: odds ratio, 95% CI: 95% confidence interval

In the choice tests, a similar trend was observed with ASB station v1.2.1 outcompeting the other bait stations. When comparing the most attractive station, ASB v1.2.1, to the least attractive one, ASB v1.1.1, 55.4% (95% CI 45.0–66.0%) of recaptured mosquitoes were attracted to ASB v1.2.1 and 44.6% (95% CI 38.1–52.6%) were attracted to ASBv1.1.1 (Fig. 5).

No difference was seen in the response between *An. gambiae* s.s. and *An. funestus* s.s. or between sexes when all treatment arms were combined; however, when investigating the impact of sex for different bait stations, slightly more male than female mosquitoes were attracted to ASB station v1.2.1 (OR = 1.19, 95% CI 1.02–1.39, *P* = 0.025) (Table 5 SOM 1).

### Determination of the intrinsic feeding success of mosquitoes on different prototypes of ASB stations

**Fig. 6:**
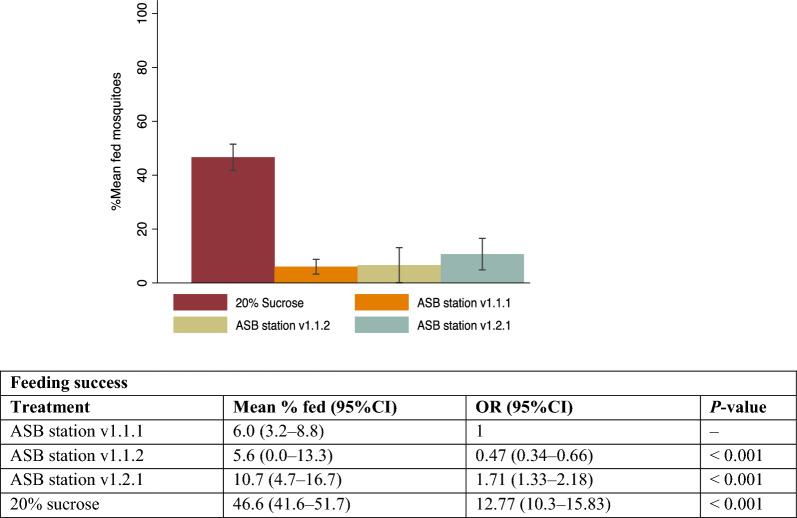
Intrinsic feeding success of ASB stations and sucrose for 3–5-day-old mosquitoes in 2 × 5 × 2-m cages. The table below provides the odds ratios (ORs) and *P*-values comparing feeding success of the mosquitoes to the bait stations and standard comparator (20% sucrose). OR: odds ratio, 95% CI: 95% confidence interval

In line with the intrinsic attraction results, recaptured mosquitoes showed higher feeding success on ASB station v1.2.1 compared to other older versions of the ASB station (Fig. 6). Feeding success on ASB station v1.2.1 was almost twice that of ASB station v1.1.1, 10.7% (95% CI 4.7–16.7%) compared to 6.0% (95% CI 3.2–8.8%) respectively (OR = 1.71, 95% CI 1.33–2.18, *P* < 0.001) but < 20% sucrose (OR = 12.77, 95% CI 10.3–15.83, *P* < 0.001; Fig. 6). Male mosquitoes showed a significantly greater proportion of feeding when responses from all ASB stations were combined (OR = 1.34, 95% CI 1.17–1.52, *P* < 0.001; Table 6 SOM 1). When stratified by species and sex, there was no significant difference in feeding success between the two species for either ASB station (Table 7 SOM 1) except for 20% sucrose where male *An. funestus* showed higher feeding success (OR = 1.51, 95% CI 1.13–2.01, *P* = 0.005) than male *An. gambiae *s.s. (OR = 1; Table 7 SOM 1).

To assess whether the gauze could be used for feeding success assessment assays as well, preliminary studies compared feeding success of *An. gambiae *s.s. and *An. funestus* mosquitoes in the presence or absence of the gauze fitted at 2 mm above the bait. The gauze reduced feeding success but did not prevent from feeding completely (Table 8 SOM 1).

#### Development of a standard operating procedure for evaluation of ASBs in a semi-field system

Based on the experiments conducted, a standard operating procedure (SOP) to evaluate mosquito attraction (intrinsic and relative attraction) to, and feeding success on, ATSB in the semi-field system was developed (SOM 2).

## Discussion

### Mosquito response (attraction) in no-choice and choice experiments

The study presented here demonstrates the importance of choice and no-choice when comparing ATSB products or sugar sources as also found when evaluating other behavioural modifiers such as topical repellents [56]. Whilst the intrinsic attraction gives an indication of the impact of a bait station when deployed alone (often the maximal impact), choice experiments may represent a more realistic situation, where sugar sources compete. The intrinsic attraction (no-choice) and relative attraction experiments showed an improved attraction to each iteration of the bait stations, and each new iteration was more attractive than the previous iterations. However, the mean percent attraction of each station was lowered when deployed in competition. There was a consistent attraction and feeding superiority of the latest iteration, ASB station v1.2.1, in both no-choice and choice experiments. This finding was replicated in a semi-field study of relative attraction of ASBs in Kenya [57]. However, the mosquitoes demonstrated lower feeding on the ASB station v1.1.2 compared to the previous prototype, ASB station v1.1.1. The modification made on ASB station v1.1.2 to reduce membrane leaking might have been the underlining factor for low feeding success.

### Response (attraction) of mosquitoes of different ages

While we hypothesised that newly emerged mosquitoes would be more attracted to sugar, based on previous work [58], the experiments showed that slightly older mosquitoes were more attracted to sugar sources than newly emerged ones, and the sugar source influenced the magnitude of difference in attraction. The difference in response between older and newly emerged mosquitoes was less pronounced for 20% sucrose than for the bait stations. This may indicate that the attractants in the ASB stations stimulate attraction of older mosquitoes more than younger ones. The observed greater attraction of the older mosquitoes to ASBs would be advantageous when using ATSBs because older mosquitoes are more likely to be malaria infected. Targeting the portion of the mosquito population responsible for transmission rather than newly emerged mosquitoes may be more efficacious in reducing malaria transmission. This finding also indicates that standardising the age of mosquitoes used in experiments is important. Based on the present results, we used 3–5-day-old mosquitoes for the following experiments, and we would recommend that 3–5-day-old mosquitoes are used for sugar bait experiments. Reducing heterogeneity in bioassays is critical, so having a large sample size per replicate will allow for a more precise estimate of the feeding rate [59].

### Response (intrinsic and relative attraction) and feeding success of male and female mosquitoes

Both male and female *An. gambiae *s.s. and *An. funestus *s.s. were shown to respond to introduced sugar meals including the Westham ASB stations. Male and female mosquitoes of multiple genera require a carbohydrate energy source shortly after emergence and then regularly for their daily activities of flight, mating, fecundity, oviposition and metabolism [20, 21]. Previous work has shown that, upon emergence, female *An. gambiae *s.s. are more attracted to honey volatiles than human odour [58].

No consistent differences in the attraction and feeding success of male and female mosquitoes were evident in our study; however, previous studies have demonstrated differing frequency of male and female sugar feeding [60] and responses of sexes to ATSBs [61]. We recommend that sugar bait bioassays use both male and female mosquitoes given that the stations aim to reduce both and both sexes feed on similar sugar sources in nature [62]. The responsiveness of both sexes is significant for vector control; a greater impact on mosquito populations could occur if both sexes are affected [22] than for interventions that target only female mosquitoes.

### Response (attraction) of different mosquito species

There was not a large difference in the responses of *An. gambiae *s.s. and *An. funestus *s.s. to the baits used in this study. This is reassuring because these highly efficient malaria vectors are sympatric throughout much of sub-Saharan Africa [63], which results in extremely high malaria transmission where they overlap [64]. There is, however, evidence of different sugar preferences among different species [20] and differential responses of *Anopheles* species to ATSB stations [57]. Even though the two studies looked at the response of the same mosquito species to the same ATSB, the difference observed is probably due to different experimental designs, experiment sites and methods used to assess the attraction.

Therefore, in line with WHO guidance for evaluating vector control products with a behavioural component, such as insect repellents [65] and household insecticidal products like volatile emanators and mosquito coils [66], it will be important to test sugar baits against representative mosquito species for a particular area. Using representative species is also recommended for interventions with an insecticidal mode of action, including ITNs [67] and IRS [68], because of species differences in responses to insecticides related to resistance mechanisms [69]. However, as *An. gambiae *s.s. and *An. funestus *s.s. share a niche, where they are highly synanthropic, they may have both developed common sugar feeding preferences for plant species associated with human habitation throughout Africa. There is evidence that *An. gambiae *s.s. has a preference for several plants commonly found near homes [62, 70] and that are widespread throughout sub-Saharan Africa including mango fruit [36], mango flowers [35] and castor plant [70]. *Anopheles funestus* and *An. arabiensis* have also been shown to demonstrate a preference for mango flowers [57]. Further studies on the sugar-feeding preferences of Afrotropical *Anopheles* are warranted to optimize the olfactory profile of bait stations for malaria control using either traps to measure response to possible candidates in the field [38, 40] or semi-field systems [38, 57] or, ideally, identifying plants in the field that wild mosquitoes have fed upon by using molecular methods to identify the main sources of sugar meals [70].

### Bioassay design

Data collected in this study agreed with another study, where mosquitoes preferentially selected sucrose over fructose [71]. However, in the current study, a no-choice assay was used to select the intrinsic attraction of the sucrose or fructose baits, whereas Kessler et al. [70] used a choice test with 292 mM sucrose versus 584 mM fructose [71]. Also, our study looked at attraction, while the former study investigated mosquito palatability. In this study, the mosquitoes' response/attraction to sugar meals was observed to increase with increased sugar concentration, highlighting that mosquitoes are likely to select sugar meals based on olfactory cues that provide information on the nutrition of the sugar source [35]. The 20% sucrose used in this study was raw cane sugar, which may contain volatile impurities, water vapour and microbial growth, which increase the attractiveness of mosquito sugar sources even at a short distance. Therefore, as this potentially introduced a bias, the final experiments compared the relative attraction of the more standardised bait stations without the inclusion of a sucrose comparator. A potential alternative or additional explanation for the apparent high level of olfactory attraction of the sucrose in our experiments is that there may have been repeat visits stimulated by its relatively high palatability, given the possibility of feeding through the electrostatic gauze. This may have also affected the attraction to different bait station versions, with the higher feeding success on v1.2.1 resulting in more revisits and increased likelihood of being heavily marked. Moreover, the mosquitoes used in these experiments are reared in an insectary where they receive sucrose ad libitum for colony maintenance; therefore, it is likely that they have been selected for a preference for sucrose, thus leading to higher attraction and feeding than any of the ASB stations. For future products using a sugar source of floral odour of plants that mosquitoes regularly feed on, for example, tropical fruit [72] or key volatiles in them [73], may improve attraction.

Mosquitoes use olfactory cues to discriminate between diverse species of plant sugar sources in their environment [40] based on beneficial volatile organic compounds [70]. Some cues are species-specific, but *An. gambiae *s.l. use both β-myrcene and (E)-β-ocimene as primary chemicals to determine whether plants are a potential nutrient source [70]. As with host cues, the response of mosquitoes to these volatiles is dose-dependent [36, 74]. Identification of optimal cues, the correct concentrations and possibly the development of a synthetic attractant blend [73] for use in ATSB formulations could increase their efficacy, especially in areas with many competing sources of sugar. Field applications of ATSB have shown a significant density reduction of several mosquito species [40, 75, 76] with very high impact evident in areas with low availability of natural sugar sources [46]. This indicates competition between bait stations and natural sugar sources is an important factor to be considered in ATSB effectiveness.

### Optimal placement of ATSB for vector control

The role of host odour in the attraction of anthropophilic mosquitoes to sites, where they may sugar feed before swarming [77] close to houses [78–80], is not known. However, it has been observed that mosquitoes will sugar feed opportunistically when a blood host cannot be accessed, for example, because of the presence of bed nets [27]. Deploying Westham ATSBs on the outer walls of a house may therefore attract the mosquitoes to rest and consequently feed on them as they approach the house for host-seeking or after taking a blood meal. *Anopheline* mosquitoes like to rest on dark surfaces [81]; therefore, deploying black-coloured sugar baits in or outside of houses may increase the chance of mosquitoes encountering the bait station, especially in areas with high bednet use [27].

Sugar feeding can reduce the mosquitoes' probability of blood feeding [82] and potential overall longevity, thus reducing vectorial capacity [83] even without the addition of a toxin. Mosquitoes often take multiple sugar meals throughout their lifetimes to supplement energy reserves [84]; therefore, providing multiple opportunities of contact to toxins incorporated in the bait. The potential impact of deploying ATSBs has been modelled [85, 86]; however, understanding how frequently mosquitoes feed on sugar (and therefore potentially on an ATSB) daily while resting/ovipositing, mating and during host-seeking is a critical parameter. If mosquitoes feed daily on sugar, there is potential for a large impact on mosquito populations using ATSBs, provided they are sufficiently attractive over a distance and placed at sufficient density to outcompete natural sugar sources.

Our studies were conducted in 2 × 5 × 2-m cages, which sets a limit on the radius of attraction tested. The volume of the arena in which the bait stations are tested is an important consideration if longer-range attraction is to be evaluated. For the optimal sugar bait, the attraction of mosquitoes at a distance is a desired characteristic [87]. There is a trade-off, however, with ensuring high recapture within a semi-field system and cage size; locating and collecting mosquitoes are more challenging in larger cages. Therefore, based on the observations of this study, we recommend conducting high-throughput bioassays in smaller cages using no-choice assays followed by choice tests with an optimal dose and design, comparing bait station prototypes against each other or with competing attractants such as the mosquitoes’ preferred natural sugar source [57]. Larger arenas for studies of attraction at a distance (again using non-choice followed by choice assays) may be a second stage for evaluation as they require more resources and are lower throughput than small cage assays. An additional consideration is whether to include the presence of blood meal hosts. Depending on how sugar baits may be envisioned to be deployed in a field setting, competition or ‘interference’ from host odours or alternative sugar sources may impact an ATSB’s attraction. This staged approach was used in developing odour baits for *An. gambiae* in SFS and then tested under field conditions with and without competition from human hosts [88].

For measuring mosquito attraction at a distance in a second stage of evaluations, using a larger square chamber would allow mosquitoes to orientate and disperse more freely in both no-choice and choice experiments. The radius of attraction could also be determined through mark-release-recapture experiments [89] with differentially marked mosquitoes released at different distances from the baits or by using sticky traps set at different distances from the point of release [40]. Alternatively, experiments using taxis boxes could measure directional movement towards bait stations [90].

### Development of an optimal standard operating procedure

The experiments conducted in this study led to the development of an SOP for evaluating anopheline attractancy, feeding and mortality against ATSBs in semi-field systems as described in the supplementary online materials (SOM 2). The SOP highlights important aspects that need to be considered when evaluating ATSBs and provides a standardized method that can be replicated at different sites allowing for comparability of findings.

### Study limitations

Our work focused on the attraction of mosquitoes to bait stations based on olfactory cues using a method to mark mosquitoes that came in close contact with the bait stations. However, using a surface marking method to assess the mosquito response/attraction may have underestimated the attractiveness of the bait if mosquitoes that visited the sugar source once were not as clearly marked as those that were repeatedly attracted. The clear marking of mosquitoes may not only measure the olfactory attraction but could also relate to palatability; repeated visits are more likely when the source is more palatable. Therefore, the intrinsic and relative attraction results presented here could have been influenced by the relative palatability of the baits, whereas the feeding assessments represent a combination of attraction and palatability of each bait. Further studies are required to determine whether the marking of mosquitoes from a single visit to the gauze is sufficient for detection. If single-visit-marked mosquitoes can be easily detected, then repeated visits would not result in an overall difference in the proportion marked, and it could be concluded that the results were not influenced by palatability. The positioning of the electrostatic gauze in front of the baits may interfere with odour plumes, thus reducing potential attractancy. Therefore, we recommend further studies to assess any differences in olfactory attraction success when the electrostatic gauze is positioned proximal versus slightly away from the bait. Our studies did not assess attractancy and feeding within the same assay and so we are unsure whether the attractancy of mosquitoes was indicative of potential feeding success. Attraction to ASBs or ATSBs may be used in assays as a proxy indication of bait station palatability and ultimately mosquito feeding success and may be used for product optimisation. In the current setup, the gauze fitted at 2 mm above the bait did reduce feeding success but did not prevent it completely. However, cues for attraction may differ to gustatory cues, so further work is needed to optimise an assay that can measure both attraction and palatability in a single assay. It is likely that placing the gauze closer to the bait would improve feeding.

Furthermore, the toxic effect of the active ingredient was not assessed in our study. To fully evaluate the efficacy of ATSBs, their attractancy, palatability and ultimately toxicity all need to be assessed. A simple method to do so is provided in the SOP (SOM 2) by scoring mosquitoes as dead or alive and marked or not on collection. However, designing an assay, where all three outcomes can be assessed simultaneously, reliably, and be assumed to be related can be challenging. The detection of surface-marked mosquitoes and of imbibed sugar meals can result in inconsistent results because of the variability in intensity of marking (as mentioned, repeat visits vs single and smaller vs larger sugar meals) even in recently attracted and fed mosquitoes. For those that may have been contacted to the sugar bait and fed at some extended period of time prior to collection and subsequently died, detecting marked mosquitoes can be more challenging; the surface marking may have rubbed off and sugar meals digested. Additionally, in this study only ASB prototype version 1.1.1 was used when determining the effect of mosquito age on the attraction to the ASB. We recommend future studies investigate the effect of mosquito age on the attraction to all three ASB prototype versions tested in the subsequent experiments in this study.

## Conclusions

This study has provided further evidence that ATSBs can be used to exploit the essential sugar-feeding behaviour to control mosquito vectors. Based on the experiments conducted in this study, we recommend that future assays to evaluate ATSB attraction and feeding should be performed using (i) 3–5-day-old mosquitoes, (ii) both sexes and (iii) multiple species. Different mosquito species and both sexes can be released in the same cage if mosquito densities within the cage are maintained to allow for natural mosquito dispersal without interference. Subsequent statistical analyses should be carried out to determine differential responses of species and sexes. The cage design is important to maximise mosquito recapture, allowing for the generation of high-throughput data. As such, we recommend constructing smaller cages that still support natural flight within a semi-field system. Data from such experiments showed that the Westham ASB station v1.2.1 elicited the highest olfactory attraction and feeding response in Anopheline mosquitoes and was the most appropriate prototype of those stations tested to be taken forward for epidemiological evaluations of public health impact.

## Supplementary Information

Supplementary Material 1.

Supplementary Material 2.

## Data Availability

This study has lead to development of SOP that may be used to assess ASB or ATSB attractant across different studies.

## Abbreviations

ASB: Attractive sugar bait
ATSB: Attractive toxic sugar bait/attractive targeted sugar bait
WHO: World Health Organisation
SOP: Standard operating procedures
SFS: Semi field system
ITN: Insecticide-treated net
IRS: Indoor residual spray
IRM: Insecticide-resistance management
SOM: Supplementary online materials
EN: Electrostatic netting
IVCC: Innovative Vector Control Consortium
