# Supplementary material for: Bioassays for the evaluation of the attractiveness of attractive targeted sugar bait (ATSB) against Anopheles mosquitoes in controlled semi-field systems
Source: Parasit Vectors. 2025 Feb 4;18:38. doi: 10.1186/s13071-024-06653-3 (PMC11792329; doi:10.1186/s13071-024-06653-3)
Supplement: Supplementary file 1 — Supplementary Material 1. [file 13071_2024_6653_MOESM1_ESM.docx]

**Supplemental Online Material (SOM 1)**

**Determination of standard comparator**

The standard comparator used in this study was determined through a series of no-choice experiments comparing 5%, 10%, 15% and 20% mass by volume (m/v) concentrations of sucrose (brown sugar produced by a Kilombero sugar company in Tanzania) or fructose (D-fructose produced by central Drug House (p) Ltd) in a solution. The prepared sugar solutions were labelled with 0.5% v/v food dye (Carmoisin) for visualization of fed mosquitoes. A guava juice (positive control) and plain water (negative control) were also used when determining the standard comparator only. Guava juice was prepared by grinding ripened guava fruits and diluting with deionized water at 3:2 v/v. All comparators (sucrose, fructose and guava juice) were prepared within two hours before starting each replicate of the experiment to minimise the microbial activity prior to the experiment.

For each treatment, six Petri dishes (Sigma Aldridge®) of an approximately 11cm diameter and 90ml volume were prepared. A foam disc was prepared from locally bought cellulose dish-washing sponge (O-Cel-O, Scotch Brite) and dipped into the freshly prepared bait solution and thereafter pressed into each Petri dish. The Petri dishes were placed on the bait delivering station (Figure 1 in main paper), overlaid with one layer of cling film, and the film was pierced in a 0.5cm grid with sterile pins to allow mosquitoes access to the sugar without excessive amounts leaking onto the surface of the cling film. Electrostatic netting with fluorescent powder was suspended 2mm by clamping with pegs in front of the cling film in order to mark any mosquitoes attempting to make contact with the standard comparators.

Experiments to evaluate olfactory attraction towards these different sucrose and fructose concentrations were carried out using 2m x 5m x 2m netted cages within chambers of a semi-field system [1]. Ten cages were prepared the morning of the experiments, eight for each of the sugar concentrations, and two for the controls. In each cage, one sugar station of a given concentration was placed at the centre of the cage. Twenty, 0 to 1-day-old, sugar- and blood-naïve female and sugar-naïve male *An. gambiae* were released into each cage at 18:00 hrs and collected the following morning at 06:00 hrs. The experiment was replicated eight times.

**Results**

Mosquito attraction to all sugar solutions, measured as percentage of the recovered mosquitoes that had fluorescent powder on their cuticle, was greater than 80% and higher than their response to distilled water (78%) or fresh guava juice (79%), regardless of mosquito species or sex. Sucrose and fructose at 20% were the most attractive sugar concentrations with more than 90% of mosquitoes landing on them. The two concentrations did not differ in attraction from each other (Table 1 SOM 1). When all the concentrations of sucrose and fructose were combined, it was seen that overall, there was no difference in response to sucrose or fructose (sucrose vs fructose OR=1.13, 95%CI: 0.90-1.42, p=0.299; Table 1 SOM 1). 20% sucrose was selected as the standard comparator because it is cheap and widely available.

|  | **Attracted mosquitoes** | | |
| --- | --- | --- | --- |
|  | **%Mean (95%CI)** | **OR (95%CI)** | **p-value** |
| ***Treatment*** | | | |
| Plain water | 78.2 (65.9-90.5) | 1 ^a^ | - |
| Guava juice | 79.4 (62.7-96.0) | 1.22 (0.83-1.79)^a,b,c^ | 0.32 |
| 5% fructose | 86.0 (73.9-98.1) | 1.80 (1.20-2.69) ^c,d^ | 0.01 |
| 5% sucrose | 80.1 (68.2-92.1) | 1.13 (0.76-1.69) ^a,b^ | 0.54 |
| 10% fructose | 92.3 (85.1-99.4) | 3.39 (2.11-5.46) ^e,f^ | <0.001 |
| 10% sucrose | 85.0 (73.9-96.2) | 1.62 (1.08-2.42)^b,c,d^ | 0.02 |
| 15% fructose | 83.2 (70.0-96.3) | 1.38 (0.93-2.06)^a,b,c^ | 0.11 |
| 15% sucrose | 88.6 (77.1-100) | 2.21(1.44-3.40)^c,d,e^ | <0.001 |
| 20% fructose | 92.0 (86.7-97.4) | 3.37 (2.11-5.36) ^e,f^ | <0.001 |
| 20% sucrose | 93.4 (87.6-99.3) | 4.54 (2.73-7.57) ^f^ | <0.001 |
|  | | | |
| ***All concentrations of either sucrose or fructose combined*** | | | |
| Sucrose | 86.8(81.5-92.1) | 1 | - |
| Fructose | 88.4(83.4-93.4) | 1.13(0.90-1.42) | 0.299 |

Table 1 SOM 1. Response of 0-1-day old *An. gambiae s.s.* to different concentrations of fructose and sucrose in no-choice tests in 2m x 5m x 2m cages.

*Differing superscript letters are significantly different from each other. Same superscript letters are not significantly different from each other*

**Determination of bait station version for experiment to assess impact of mosquito age on attractiveness of attractive sugar bait stations**

Newly emerged mosquitoes were exposed in four separate 2m x 5m x 2m cages in the SFS to either ASB station v1.0 (with bait), a blank ASB station v1.1.1 (ASB without attractant/bait), ASB station v1.1.1 (with bait), or 20% sucrose. Fifty, 0 to 1-day-old male and female, sugar and blood-naïve *An. gambiae s.s.* were released into the cages each night from 18:00 hours and collected 12 hours later in the morning. The experiment was replicated for eight experimental nights. In a second experimental setup, one cage of the same dimensions was used with three stations set up within it, ASB station v1.0, the blank ASB station v1.1.1 and ASB station v1.1.1 (with bait) to assess relative attraction. Similarly, 50, 0 to 1-day-old male and female sugar- and blood-naïve *An. gambiae s.s.* were exposed for 12 hours, and the experiment was replicated eight times.

**Results**

Intrinsic attraction of 0 to 1-day-old *An. gambiae s.s.* was greatest for 20% sucrose 60.4% (95%CI: 49.2-71.6) and least for the blank ASB station v1.1.1 15.4% (95% CI: 7.6-23.2). ASB station v1.1.1 was more attractive than the previous version ASB station v1.0,19.6% (95% CI: 9.9-29.4) vs 25.9% (95%CI: 18.5-33.4; OR 1.35, 95% CI: 1.02-1.79, p=0.033; SOM Table 2 and SOM 1). Similarly, in the choice experiment to assess relative attraction, mosquitoes were more attracted to ASB station v1.1.1 64.6%, (95% CI: 45.1-73.5) than the ASB station v1.0, 25.3%, (95% CI: 13.9-36.7) and the blank station 10.0% (95% CI: 0.8-19.3; Table 2 and SOM 1).

|  | **Intrinsic attraction** | | | **Relative attraction** | | |
| --- | --- | --- | --- | --- | --- | --- |
| ***Treatment (dose)*** | **%Mean attracted (95%CI)** | **OR (95% CI)** | **p-value** | **%Mean attracted (95%CI)** | **OR (95%CI)** | **p-value** |
| ASB station v1.0 | 19.6(9.9-29.4) | 1^a^ | - | 25.3(13.9-36.7) | 1^a^ | - |
| ASB station v1.1 blank | 15.4(7.6-23.2) | 0.71(0.52-0.97)^b^ | 0.034 | 10.1(0.8-19.3) | 0.44(0.25-0.78)^b^ | 0.005 |
| ASB station v1.1.1 | 25.9(18.5-33.4) | 1.35(1.02-1.79)^c^ | 0.033 | 64.6(45.1-73.5) | 3.19 (2.15-4.82)^c^ | <0.001 |
| 20% sucrose | 60.4(49.2-71.6) | 5.79(4.42-7.58)^d^ | <0.001 | - | - | - |

Table 2 SOM 1. Intrinsic and relative attraction of different prototypes of ASB stations and sugar against 0 to 1-day-old *An. gambiae*

*Differing superscript letters are significantly different from each other. Same superscript letters are not significantly different from each other.*

|  | **Mean % attraction (95% CI) to ASB v.1.1.1.** | **OR (95%CI)** | **p-value** | **Mean % attraction (95% CI) to 20% sucrose** | **OR (95%CI)** | **p-value** |
| --- | --- | --- | --- | --- | --- | --- |
| **Female** | | | | | | |
| 0 1-day-old | 27.7(14.5-40.8) | 1 | - | 63.2(46.1-80.2) | 1 |  |
| 3 to5-day-old | 77.3(69.6-85.0) | 9.12(6.62-12.55) | <0.001 | 86.4(79.8-93.1) | 3.91(2.82-5.44) | <0.001 |
| **Male** | | | | | | |
| 0 to 1-day-old | 24.2(14.9-33.5) | 1 |  | 57.6(40.3-74.8) | 1 |  |
| 3 to 5-day-old | 72.3(63.5-81.2) | 7.96(5.72-11.09) | <0.001 | 88.1(83.2-92.9) | 5.45(3.94-7.53) | <0.001 |

Table 3 SOM 1. The intrinsic attraction of 0 to 1-day-old compared to 3 to 5-day-old *Anopheles gambiae s.s.* when exposed to either ASB v1.1.1 (Westham bait station) or 20% sucrose investigating impact of mosquito sex on response.

|  | **Intrinsic attraction** | | |
| --- | --- | --- | --- |
| **Treatment** | **Mean % attraction (95% CI) attracted** | **OR (95%CI)** | **p-value** |
| ASB station v1.1.1 | 83.2(80.2-86.1) | 1^a^ | - |
| ASB station v1.1.2 | 77.5(73.5-81.5) | 0.74(0.66-0.84)^b^ | <0.001 |
| ASB station v1.2.1 | 91.2(87.9-94.5) | 2.11(1.79-2.49)^c^ | <0.001 |
| 20% sucrose | 91.8(89.9-93.7) | 2.32(2.05- 2.62)^c^ | <0.001 |
| **Mosquito species** |  |  |  |
| *Anopheles gambiae* s.s. | 87.1(85.1-89.2) | 1 | - |
| *Anopheles funestus* | 86.2(83.9-88.5) | 0.93(0.84-1.02) | 0.132 |
| **Mosquito sex** |  |  |  |
| *Female* | 87.2(85.0-89.4) | 1 | - |
| *Male* | 86.1(83.9-88.3) | 0.87(0.79-0.95) | 0.094 |
| **Treatment and mosquito species interaction** |  |  |  |
| ***ASB station v1.1.1*** |  |  |  |
| *Anopheles gambiae* s.s. | 83.6(79.5-87.7) | 1 | - |
| *Anopheles funestus* | 82.7(78.2-87.1) | 0.95(0.82-1.11) | 0.514 |
| ***ASB station v1.1.2*** |  |  |  |
| *Anopheles gambiae* s.s. | 77.9(72.3-83.5) | 1 | - |
| *Anopheles funestus* | 77.2(71.1-83.3) | 0.97(0.80-1.18) | 0.739 |
| ***ASB station v1.2.1*** |  |  |  |
| *Anopheles gambiae* s.s. | 92.4(89.3-95.5) | 1 | - |
| *Anopheles funestus* | 89.9(83.9-96.0) | 0.70(0.52-0.94) | 0.018 |
| ***20% sucrose*** |  |  |  |
| *Anopheles gambiae* s.s. | 92.0(89.3-94.6) | 1 | - |
| *Anopheles funestus* | 91.6(88.9-94.4) | 0.97(0.80-1.18) | 0.787 |
| **Treatment and Mosquito sex interaction** |  |  |  |
| ***ASB station v1.1.1*** |  |  |  |
| *Female* | 83.5(79.2-87.9) | 1 | - |
| *Male* | 82.8(78.5-87.0) | 0.89(0.76-1.04) | 0.128 |
| ***ASB station v1.1.2*** |  |  |  |
| *Female* | 80.8(75.7-86.0) | 1 | - |
| *Male* | 74.3(68.1-80.5) | 0.67(0.55-0.82) | <0.001 |
| ***ASB station v1.2.1*** |  |  |  |
| *Female* | 91.2(85.8-96.5) | 1 | - |
| *Male* | 91.3(86.9-95.4) | 1.03(0.77-1.39) | 0.820 |
| ***20% sucrose*** |  |  |  |
| *Female* | 91.6(88.6-94.6) | 1 | - |
| *Male* | 91.5(89.6-94.4) | 0.99(0.81-1.21) | 0.926 |

Table 4 SOM 1. The intrinsic attraction of Westham ASB stations against 3 to 5-day-old *An. gambiae* and *An. funestus*

Table 5 SOM 1. Relative attraction of different ASB stations in choice tests against 3 to 5-day-old *An. gambiae* and *An. funestus* in 2m x 5m x 2m cages investigating impact of mosquito sex and species on response

|  | **Mean % attraction (95% CI)** | **OR (95%CI)** | **p-value** |
| --- | --- | --- | --- |
| ***Mosquito species for all arms*** |  |  |  |
| *An. gambiae* | 21.3(18.5-24.2) | 1 | - |
| *An. funestus* | 23.2(19.4-27.0) | 1.12(1.03-1.23) | 0.080 |
| ***Mosquito sex for all arms*** |  |  |  |
| Female | 21.7(18.4-25.1) | 1 |  |
| Male | 22.8(19.4-26.2) | 1.04(0.96-1.14) | 0.320 |
| **Treatment and mosquito species interaction** |  |  |  |
| ***ASB station v1.1.1*** |  |  |  |
| *Anopheles gambiae* s.s. | 18.0(14.8-21.3) | 1 | - |
| *Anopheles funestus* | 19.7(15.7-23.8) | 1.12(0.98-1.27) | 0.102 |
| ***ASB station v1.1.2*** |  |  |  |
| *Anopheles gambiae* s.s. | 17.8(13.5-22.1) | 1 | - |
| *Anopheles funestus* | 19.7(11.8-27.6) | 1.12(0.92-1.36) | 0.251 |
| ***ASB station v1.2.1*** |  |  |  |
| *Anopheles gambiae* s.s. | 29.7(22.5-36.9) | 1 | - |
| *Anopheles funestus* | 32.0(22.4-41.5) | 1.15(0.99-1.34) | 0.070 |
| **Treatment and mosquito sex interaction** |  |  |  |
| ***ASB station v1.1.1*** |  |  |  |
| *Female* | 19.4(15.3-23.5) | 1 | - |
| *Male* | 18.4(15.1-21.7) | 0.89(0.79-1.02) | 0.096 |
| ***ASB station v1.1.2*** |  |  |  |
| *Female* | 17.7(12.3-23.1) | 1 | - |
| *Male* | 19.7(12.6-26.9) | 1.20(0.99-1.46) | 0.070 |
| ***ASB station v1.2.1*** |  |  |  |
| *Female* | 28.8(20.4-37.2) | 1 | - |
| *Male* | 32.9(24.4-41.4) | 1.19(1.02-1.39) | 0.025 |

Table 6 SOM 1. Feeding success of different ASB stations against 3 to 5-day-old *An. gambiae* and *An. funestus* in 2m x 5m x 2m cages, investigating impact of mosquito sex and species on response

|  | **Mean % fed(95% CI)** | **OR (95%CI)** | **p-value** |
| --- | --- | --- | --- |
| ***Mosquito sex for all arms*** |  |  |  |
| *Female* | 14.8(9.8-19.9) | 1 | - |
| *Male* | 20.2(14.1-26.2) | 1.34(1.17-1.52) | <0.001 |
| ***Mosquito species for all arms*** |  |  |  |
| *Anopheles gambiae* s.s. | 15.6(10.6-20.6) | 1 | - |
| *Anopheles funestus* | 19.4(13.2-25.5) | 1.22(0.84-1.79) | 0.296 |
| ***Treatment and mosquito species interaction*** | | | |
| ***ASB station v1.1.1*** |  |  |  |
| *Anopheles gambiae* s.s. | 4.6(1.0-8.1) | 1 | - |
| *Anopheles funestus* | 7.5(2.8-12.2) | 1.22(0.83-1.79) | 0.300 |
| ***ASB station v1.1.2*** |  |  |  |
| *Anopheles gambiae* s.s. | 7.3(0.0-22.2) | 1 | - |
| *Anopheles funestus* | 4.2(0.0-9.2) | 0.99(0.57-1.74) | 0.980 |
| ***ASB station v1.2.1*** |  |  |  |
| *Anopheles gambiae* s.s. | 10.0(0.8-22.0) | 1 | - |
| *Anopheles funestus* | 11.4(2.9-17.1) | 0.94(0.69-1.30) | 0.723 |
| ***20% sucrose*** |  |  |  |
| *Anopheles gambiae* s.s. | 42.3(36.8-47.7) | 1 | - |
| *Anopheles funestus* | 51.0(42.5-59.5) | 1.25(1.03-1.53) | 0.025 |
| ***Treatment and mosquito sex interaction*** | | | |
| ***ASB station v1.1.1*** |  |  |  |
| *Female* | 4.9(0.6-9.1) | 1 | - |
| *Male* | 7.2(3.1-11.3) | 1.36(0.92-1.99) | 0.121 |
| ***ASB station v1.1.2*** |  |  |  |
| *Female* | 3.4(0.1-6.6) | 1 | - |
| *Male* | 9.8(0.0-23.4) | 1.92(1.07-3.44) | 0.028 |
| ***ASB station v1.2.1*** |  |  |  |
| *Female* | 8.5(5.3-21.6) | 1 | - |
| *Male* | 5.7(0.5-11.0) | 0.94(0.69-1.30) | 0.723 |
| ***20% sucrose*** |  |  |  |
| *Female* | 45.4(37.9-52.8) | 1 | - |
| *Male* | 47.9(40.3-55.5) | 1.25(1.03-1.53) | 0.024 |

| **Treatment** | **Female** | | | **Male** | | | |
| --- | --- | --- | --- | --- | --- | --- | --- |
|  | **Mean percentage fed** | **OR (95%CI)** | **p-value** | **Mean percentage fed** | **OR (95%CI)** | | **p-value** |
| ASB station v1.1.1 | 4.9(0.6-9.1) | 1 | - | 7.2(3.1-11.3) | 1 | | - |
| ASB station v1.1.2 | 3.4(0.1-6.6) | 0.38(0.22-0.66) | 0.001 | 9.8(0.0-23.4) | 0.54(0.35-0.82) | | 0.004 |
| ASB station v1.2.1 | 5.7(0.5-11.0) | 1.10(0.74-1.65) | 0.631 | 15.7(4.7-26.7) | 2.25(1.64-3.08) | | <0.001 |
| 20% sucrose | 45.4(37.9-52.8) | 13.0(9.40-18.0) | <0.001 | 47.9(40.3-55.5) | 12.2(9.17-16.27) | | <0.001 |
| ***Visits to bait stations by mosquito species*** | | | | | | | |
| *Anopheles gambiae s.s.* | 13.8(7.1-20.5) | 1 | - | 17.4(9.7-25.1) | 1 | | - |
| *Anopheles funestus* | 15.9(8.0-20.5) | 0.97(0.79-1.22) | 0.853 | 22.9(13.2-32.6) | 1.32(1.08-1.61) | | 0.006 |
| ***Treatment and mosquito species interaction*** | | | | | | | |
| ***ASB station v1.1.1*** | | | | | | | |
| *Anopheles gambiae s.s.* | 3.5(0.0-9.6) | 1 | - | 5.6(0.2-10.9) | 1 |  | |
| *Anopheles funestus* | 6.2(0.0-13.6) | 1.10(0.61-1.98) | 0.751 | 8.8(1.3-16.3) | 1.32(0.80-2.17) | 0.277 | |
| ***ASB station v1.1.2*** | | | | | | | |
| *Anopheles gambiae s.s.* | 3.2(0.0-8.4) | 1 | - | 5.1(0.0-15.3) | 1 | - | |
| *Anopheles funestus* | 3.6(0.0-8.9) | 0.96(0.38-247) | 0.940 | 14.6(0.0-43.5) | 1.01(0.50-2.02) | 0.980 | |
| ***ASB station v1.2.1*** | | | | | | | |
| *Anopheles gambiae s.s.* | 5.6(0.0-13.6) | 1 | - | 17.2(0.0-39.1) | 1 | - | |
| *Anopheles funestus* | 5.8(0.0-14.9) | 0.65(0.37-1.15) | 0.138 | 14.2(1.6-26.7) | 1.13(0.77-1.66) | 0.540 | |
| ***20% sucrose*** | | | | | | | |
| *Anopheles gambiae s.s.* | 42.8(35.2-50.3) | 1 | - | 41.8(31.8-51.8) | 1 | - | |
| *Anopheles funestus* | 48.0(33.0-62.9) | 1.06(0.80-1.39) | 0.699 | 54.0(42.1-66.0) | 1.51(1.13-2.01) | 0.005 | |

Table 7 SOM 1. Percentage mean and odds ratio of mosquito feeding success on ASB stations in 2m x 5m x 2m cage.

Table 8 SOM 1. Mean percentage of fed mosquitoes when the ASB stations are overlaid or not with electrostatic gauze in 2m x 5m x 2m cage.

|  | With Electrostatic gauze | Without Electrostatic gauze |
| --- | --- | --- |
| Treatment | Mean % fed (95%CI) | Mean % fed (95%CI) |
| ASB station v1.1.1 | 1.1 (0.7-1.5) | 6.0 (3.2-8.8) |
| ASB station v1.1.2 | 1.2 (0.7-1.6) | 5.6 (0.0-13.3) |
| ASB station v1.2.1 | 1.2 (0.6-1.9) | 10.7 (4.7-16.7) |
| 20% sucrose | 35.0 (31.2-38.8) | 46.6 (41.6-51.7) |

References.

1. Massue, D.J., et al., *Comparing the new Ifakara Ambient Chamber Test with WHO cone and tunnel tests for bioefficacy and non-inferiority testing of insecticide-treated nets.* Malaria journal, 2019. **18**(1): p. 1-15.
