# Supplementary material for: Bioassays for the evaluation of the attractiveness of attractive targeted sugar bait (ATSB) against Anopheles mosquitoes in controlled semi-field systems
Source: Parasit Vectors. 2025 Feb 4;18:38. doi: 10.1186/s13071-024-06653-3 (PMC11792329; doi:10.1186/s13071-024-06653-3)
Supplement: Supplementary file 2 — Supplementary Material 2. [file 13071_2024_6653_MOESM2_ESM.docx]

| STANDARD OPERATING PROCEDURE | |
| --- | --- |
| **SOP title** | **Conduct of Attractive Targeted Sugar Bait Semi Field Evaluations (SOM 2)** |
| **SOP version.** | **v01** |
| Version date |  |
| Author/s | Frank Tenywa |
| Reviewer/s | Jenny Stevenson /Sarah Moore |
| Sponsor | IVCC |

## Purpose

This SOP describes the conduct of semi-field evaluation of Attractive Targeted Sugar Baits (ATSBs) for the evaluation of intrinsic and relative mosquito olfactory and feeding response (palatability) of ATSB prototypes at short range.

This SOP is specifically designed for studies involving the conduct of bioassays for evaluation of Westham Attractive Targeted Sugar Baits (ATSBs) and Attractive Sugar Baits without insecticide (ASBs). If ATSBs/ASBs of other designs and dimensions are to be used, the frame and dimensions of the netting would have to be adjusted accordingly.

## Training Requirements

- Laboratory safety training
- Chemical preparation training (if applicable)

## Safety

Personal protective equipment (PPE) must always be worn in the laboratory and when handling ATSB/ASB stations. This includes the use of laboratory coats and gloves for all personnel involved in this SOP.

For ATSBs containing insecticides, the material safety data sheet should be read, guidance adhered to, and the sheet filed in the storage area where the test items are located.

**PART 1. PREPARATION of STANDARD COMPARATOR BAITS**

## Equipment & Supplies

Preparation of bait stations delivery batteries

- Laboratory coat
- Laboratory gloves
- Glass beakers (1000ml capacity) (one beaker per treatment)
- Plastic buckets/ containers (1L-10L) (1-2 buckets)
- Analytical balance
- Pipette (200-1000µl)
- Wooden or steel stirring rods (the size will depend on the size of beakers used)
- Syringes (hypodermic needle) /pins (0.1-0.15mm diameter)
- Knife / scissors
- ATSB delivering frame (aluminium tray) large enough to accommodate ATSB/ASB
- Aluminium rod for stand
- Plastic basin (5-10L, approx. 0.4m diameter)
- Sand
- Untreated netting (0.5m per treatment) (can use untreated mosquito net, mesh size ~256 holes/in^2^, 2mm x 2mm
- ATSB stations and/or ASB stations (with dimensions of Westham baits)

Evaluation of baits

- Large 2 x 5 x 2m white netted cages to erect in semi-field chamber (the number of cages will depend on the number of replicates)
- Fluorescence microscope (to assess attraction and palatability)
- Light Microscope with UV torch (to assess attraction) (if a fluorescent microscope is not available)
- Prokopack aspirator
- 6-volt battery

Preparation of baits

- Disinfecting agent (ethanol, bleach)
- Laboratory gloves
- Distilled/deionised water
- Sucrose (Lab grade fructose is preferred if resources are available)
- Weigh boats (20-50 cm diameter or width)
- Pipette (200-1000µl)
- Pipette tips (1ml)
- Red food dye/yellow uranine
- Petri dishes (90ml), sterile (preferably new)
- Cellulose sponge (1.2 cm thick)
- Cling film
- Masking tape
- Aluminium foil
- Electrostatic netting (Pollentec), black. (Mosquito net gauge size, ~256 holes/in^2^, 2mm x 2mm)
- Fluorescent powder (Wtrcsv 8 colour UV fluorescent Powder, Neon UV Glow Powder Safety Pigment Powder for Paint, Slime, Nails, Resin, Concerts).
- Cotton wool
- Pegs/Clamps (clothes pegs are suitable)
- White plastic tray 30(cm x50cm) that fits the bait station for dusting electrostatic netting
- Forceps (5-10 inches)

Evaluation of baits

- Distilled water
- Sucrose
- Cotton wool
- Paper cups (250ml or larger)
- Netting (for covering paper cups and a plastic dish with sand)
- Elastic bands
- Foil/cling film
- Data forms

## Procedure

Ensure a laboratory coat and gloves are used throughout the preparation of the sucrose and ATSB/ASB batteries, and when erecting the batteries in the semi-field cages.

- 1. **Preparation of weighted base using sand**
- Fill plastic basins with sand to the level of the top of the basin.
- Wet the sand with tap water before inserting the aluminium rod to prevent the emission of dust from the sand that could affect mosquitoes.
- Insert aluminium rod into a weighted basin with base of the rod located centrally at the bottom of the basin, ensuring the sand is well compacted around it.
- Cover the weighted basin with netting to prevent mosquitoes entering the dampened sand (Figure 1d)
- Make sufficient weighted stands for all the sucrose and ATSB/ASB delivering batteries to be tested.

NB: 2.1 Alternatively the rod can be inserted in cement within the basin to form a more permanent base of cement. If step 2.1 is not feasible in your settings, use any other alternative that will make sure the bait station is standing upright and stable.

## Sanitisation and preparation of equipment and materials.

- Ensure that the laboratory workbench and relevant equipment is cleaned using ethanol/bleach and are dry prior to usage.
- Assemble all the necessary equipment and materials (as per section 1 above) on the work bench. Make sure you put on PPE before assembling the equipment and materials needed.

## Preparation of sugar solutions.

Note, preferably sugar solutions should be prepared in advance, autoclaved, and then stored in the fridge. If autoclaving is not possible, the sugar solution should be prepared on the day of each experiment to minimise bacterial growth that may affect the results.

The quantities below are for one litre of sugar solution:

- Label a 1 litre glass beaker with the sugar solution concentration to be made up.
- Weigh the appropriate amount of sugar (see Table 1 below) in a weigh boat using an analytical balance
- Pour the sugar into the beaker as shown in Table 1 for weight per volume E.g. to make 20% sugar solution, measure 200g of sugar then add water until it reaches 1000ml.
- Stir the water until the sugar is fully dissolved using a sterilised stirring rod.
- Label the beaker with the sugar used, concentration and date of preparation.

Table 1. Quantity (grams) of sugar needed to make 1000ml sugar solution for different sugar solution concentrations.

| **Sucrose / fructose** | |
| --- | --- |
| **Concentration** | **Quantity per 1000ml sugar solution** |
| 5% | 50g |
| 10% | 100g |
| 15% | 150g |
| 20% | 200g |

## Labelling sugar solutions with food dye or uranine to track feeding

**If using food dye**

- - - Using a sterilised pipette and new tip remove and discard 5ml of sugar solution from the 1 litre beaker of sugar solution prepared under 2.3.
    - Using a clean pipette tip add exactly 5ml of food dye to the remaining 995ml sugar solution (see Table 2).
    - Stir until homogenised using a clean glass stirrer and then add the words ‘food dye (0.5%)’ to the label on the beaker,
    - Make up enough “food dye” beakers for the number of sugar bait batteries to be used in the experiment.

**OR**

**If using uranine**

- - - Using an analytical balance measure 8g of uranine in a clean weigh boat
    - Pour the 8g uranine into a 1 litre beaker then add the prepared sugar solution until it reaches 1 litre (see Table 2).
    - Stir until homogenised using a clean glass stirrer and then add the words ‘uranine dye (0.8%)’ to the label on the beaker.
    - Make up enough “uranine dye” beakers for the number of sugar bait batteries to be used in the experiment.

Table 2. Quantity of food dye (ml) and uranine (g) needed per 1000ml of sugar solution.

| **Dye** | **Concentration** | **Quantity per 1000ml sugar solution** |
| --- | --- | --- |
| Food dye | 0.5% | 5ml |
| Uranine | 0.8% | 8g |

## Preparation of sugar baits and delivering battery

The quantities below are for one bait station:

- Put on clean laboratory gloves.
- Place the 6 Petri dishes on the work bench. Petri dishes should be new or sterilised if they have been used.
- Label the Petri dishes on the back with the treatment (date of experiment*, sugar type, concentration, and dye and concentration used).
- Remove new sponges from the packet and cut 6 sponges to exactly fit the Petri dishes using scissors or a knife.
- Soak the sponges into the relevant bait solutions/concentration (150-200ml per sponge) and place them in their respective Petri dishes (Figure 1). Make sure the solution is not dripping (squeeze the sponge to remove excess solution).
- Overlay each individual Petri dish with cling film ensuring the sugar solution does not leak out (change the cling film if it is leaking).
- Place the six Petri dishes into the aluminium frame to create a bait station delivery battery (see Figure 1).
- Once in the frame, use pins to pierce the cling film and make 50 pores in each Petri dish with 0.5 cm of spacing between each. These pores are needed for the mosquitoes to feed on the extruded sugar solution. A piercing tool can be constructed by pressing the pins at 0.5cm apart into a rubber (see Figure 2 of piercing tool with 0.5cm spacing between pins).
- Label the bait station delivering battery on the back of the tray with the treatment (sugar type, concentration, and concentration of dye used).
- Repeat steps above for each sugar bait station used. Ensure use of a new pins for piercing the cling film for each treatment arm to avoid contamination.

*Assuming the experiment is run on the same day as making up these dishes

## Placing the ATSB/ASB bait stations onto the frame to make a bait station delivering battery.

- Put on fresh gloves.
- Place the ATSB/ASB bait stations to be tested on the working bench.
- Label the bait station delivering battery with the version/code of the bait station being used (if multiple versions are being compared).
- If advised to do so (follow manufacturers guidelines), clean the bait station with a paper towel or cotton wool as per ‘ATSB0028_Preparing bait stations SOP_IVCC’.
- Place the cleaned bait station on the bait station frame with its black membrane side facing upward.
- Using masking tape, fix the station on the frame on all four sides (make sure there is no overlapping of the tape onto the bait station membrane).

## If assessing bait attractancy only or both attractancy and feeding: Covering the sugar and ATSB/ASB station delivering battery with electrostatic gauze net

- Put on new gloves.
- Cut a piece of electrostatic netting of equal size to the bait battery frame.
- Dust the bottom of a white plastic tray with fluorescent powder ensuring even distribution across the base of the tray such that it covers the base fully. If undertaking choice experiments, also have another tray with a different colour of fluorescent powder.
- Lay a piece of an electrostatic netting on the white plastic tray and carefully press the electrostatic netting so that the whole of one side of the net is evenly coated with the fluorescent powder from the tray. Any remaining powder can remain in the tray and be covered with foil/cling film ready to be used for coating other netting.
- Carefully cover each battery with the marked powdered electrostatic netting. The marked side of the net should be facing outwards.
- Using pegs, clamp the electrostatic netting on all side of the battery frame (Figure 1c).
- Place the bait station delivering battery on its weighted base (Figure 1d).

## Experimentation

Note, to support mosquito survival in the semi-field system and to encourage flight activity, the relative humidity (RH) in the SFS should be above 40% and temperatures between 20^0^C and 33^0^C at night throughout the course of the experiment. If available, a climatic logger should be installed in the SFS to monitor RH and temperature during the experiment.

## Assessment of mosquito attraction, feeding, and mortality (intrinsic and relative) to bait stations

**Experimental procedures in SFS**

- Clean SFS chambers as per ‘IT LN SOP 003 V02 Ifakara Ambient Chamber LN Test’, removing predators and ensuring ants cannot enter the cage to avoid scavenging of resting or dead mosquitoes.
- Prepare the large 2m x5m x2m white netted net cages, erecting them within the SFS. Ensure enough are installed for the number of treatment arms and replicates to be used.
- Cages should be completely sealed with a zip and taped to the ground to retain all mosquitoes after release.
- Place the prepared bait station delivering batteries into their respective cages according to the experimental design.

**Note**: ***For both attraction and feeding assessment studies, the batteries with the dusted electrostatic netting should be used. For assessing feeding and/or mortality only, the batteries without the netting should be deployed.***

***For the intrinsic tests (no choice), each treatment (bait station) should be placed individually in a separate cage while for the relative tests (choice), two or more treatments (bait stations) are placed into one cage at 1 metre equidistant from each other (Figure 3). Every time, ensure that cotton wool soaked in water is placed in a cage as a source of water.***

- Into each cage place a source of water for mosquitoes to remain hydrated, e.g. cotton wool soaked in deionised/distilled water placed in a small container and set on the ground 1 metre from the bait station.
- At 18:00 release approximately 50 female and 50 male 3-5 day old, blood naïve mosquitoes that have been starved for 6-8 hours of sugar prior to release. *Note: the starvation period usually depend on the mosquito species, e.g for Anopheles mosquitoes, 6-8 hours is the optimal starvation time.* Ensure equal numbers of each sex and species are released, but no more than 200 mosquitoes per cage should be used (for example, if using 2 species, 50 of each sex can be used, but if testing 3 species reduce the number per sex e.g. 30 of each sex of each species).

***Note, releases should be of morphologically distinguishable species, otherwise molecular identification methods will be needed for separation of morphologically identical species on recapture.***

- The next day at 10:00, carefully collect all mosquitoes from the cage using a Prokopack aspirator, ensuring minimal damage to mosquitoes. Collect dead mosquitoes on the floor and other surfaces first and transfer to labelled paper cups. Then collect live mosquitoes in separate cups fitted with netting covers, ensuring no more than 20-30 mosquitoes are collected into each 250 ml cup. Cups should be labelled with treatment, mosquito status (dead/alive), date of collection and cage number.
- Take all labelled cups to the insectary/lab.

***If mortality is not to be assessed:***

- Put all live mosquitoes into a freezer for 10-15 minutes to kill them.
- Assess marked status for mosquitoes (see below).
- And assess feeding status for mosquitoes (see below)

***If mortality and delayed mortality is to be determined (from feeding experiments):***

- For those mosquitoes collected alive at the end of the experiment, provide cups with cotton wool soaked with 10% w/v sugar solution and keep them at 27ºC ±2 ºC and 75% ±20% relative humidity.
- Assess abdominal marked status for dead collected mosquitoes (see below).
- Assess delayed mortality of live mosquitoes at intervals post collection as directed by study protocol (e.g. 24, 48, 72 hours etc post-collection). At each time point, remove dead mosquitoes into cups labelled with treatment, mosquito status as ‘dead at xx hour’, date of collection of experiment and cage number. Assess marked status for these dead mosquitoes, separately for each time point.
- At completion of the end of the holding time add to the label of the cup of remaining live mosquitoes, the mosquito status as ‘alive at xx hour’ post-collection.
- Place all cups of live mosquitoes into a freezer for 10-15 minutes to kill them and then assess marked status, separately for each holding time, if delayed mortality was assessed at multiple time points post-collection.

NB: Although not detailed here, live mosquitoes can be collected and held to also determine the sublethal effects of the toxin e.g. fertility, fecundity etc.

**Assessment of cuticular marking from attraction experiments**

- To assess the presence of florescent dye on the tarsi/head of mosquitoes (i.e. an indication of attraction), start with those mosquitoes that were collected dead from the SFS immediately after exposure.
- Place the mosquitoes in a Petri dish placed on top of white paper, and with the aid of a fluorescence microscope examine the mosquitoes for the presence of marking powder on their bodies. (Note if a florescence microscope is not available a light microscope mounted with a UV torch is sufficient to observe fluorescent dust on mosquitoes). Score the number of mosquitoes marked and not marked
- Repeat this for those caught alive at the end of the experiment
- For the relative attraction test, remember to record the mosquitoes by colour to distinguish which bait station they visited. If the mosquitoes went to both bait stations the mosquitoes will have more than one colour on them, and this should also be recorded in a separate category.
- Record the number marked or unmarked of those that were dead at the end of the experiment and those that were alive.

**Assessment of sugar feeding status from feeding and mortality experiments**

- To assess the mosquito feeding status, similarly, study dead/alive mosquitoes separately starting with those collected dead at the end of the experiment.
- Transfer the mosquitoes to a Petri dish and place the dish under a fluorescence microscope to observe for the presence of uranine (the fluorescent marker) within the mosquito thorax or abdomen or both, as per ‘SOP ATSB 044 Identification of mosquitoes that have fed on a uranine sugar source FINAL V1.3 (July 3, 2020)’. If present, mark as ‘fed’ on the form (see below).
- Repeat this for the mosquitoes that were held for delayed mortality assessments and read separately those that died at each mortality assessment interval to those that were alive at the end of the delayed mortality monitoring (i.e. read those dead at 24 hours separately to those that were alive at 24 hours if this was the extent of monitoring, or for example where mortality at multiple time points are assessed up to 72 hours, those dead at 24 hours separately to those that died at 48 hours to those dead at 72 hours to the remaining live mosquitoes at 72 hours).
- Record the number sugar-fed and unfed and if they are alive or dead on the data collection form.


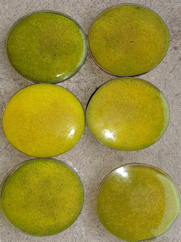

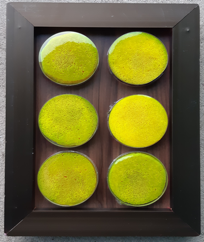

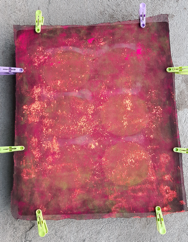

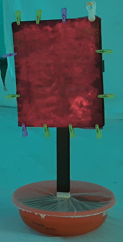


a

c

b

d

Figure 1. The battery of sugar solution filled Petri dishes. **a**) Petri dishes covered with cling film, **b**) cling film covered Petri dishes on aluminium tray **c**) Petri dishes overlaid with electrostatic black gauze with fluorescent powder, **d**) ATSB battery sitting on dish with net to prevent mosquitoes from feeding if leaking/dripping of the solution occurs.


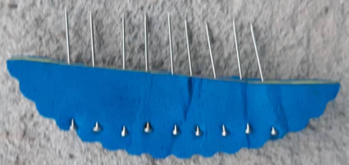


Figure 2. Cling film piercing tool with pins spaced 0.5cm apart.

Figure 3. Schematic illustration showing ATSB evaluation in the cages in the semi-field system**. a)** Cages set up for no choice tests **b)** Cages set up for choice tests **c)** Mosquitoes fed on uranine-dyed ATSB

***END OF SOP***
